# Effects of oral glucose-lowering drugs on long term outcomes in patients with diabetes mellitus following myocardial infarction not treated with emergent percutaneous coronary intervention - a retrospective nationwide cohort study

**DOI:** 10.1186/1475-2840-9-54

**Published:** 2010-09-16

**Authors:** Casper H Jørgensen, Gunnar H Gislason, Charlotte Andersson, Ole Ahlehoff, Mette Charlot, Tina K Schramm, Allan Vaag, Steen Z Abildstrøm, Christian Torp-Pedersen, Peter R Hansen

**Affiliations:** 1 Department of Cardiology, Copenhagen University Hospital Gentofte, Hellerup, Denmark; 2 Steno Diabetes Center, Gentofte, Copenhagen, Denmark; 3 Department of Cardiology, Copenhagen University Hospital Bispebjerg, Copenhagen, Denmark; 4 National Institute of Public Health, University of Southern Denmark, Copenhagen, Denmark; 5 Faculty of Health Sciences, Panum Institute, University of Copenhagen, Copenhagen, Denmark

## Abstract

**Background:**

The optimum oral pharmacological treatment of diabetes mellitus to reduce cardiovascular disease and mortality following myocardial infarction has not been established. We therefore set out to investigate the association between individual oral glucose-lowering drugs and cardiovascular outcomes following myocardial infarction in patients with diabetes mellitus not treated with emergent percutaneous coronary intervention.

**Materials and methods:**

All patients aged 30 years or older receiving glucose-lowering drugs (GLDs) and admitted with myocardial infarction (MI) not treated with emergent percutaneous coronary intervention in Denmark during 1997-2006 were identified by individual-level linkage of nationwide registries of hospitalizations and drug dispensing from pharmacies. Multivariable Cox regression models adjusted for age, sex, calendar year, comorbidity, and concomitant pharmacotherapy were used to assess differences in the composite endpoint of non-fatal MI and cardiovascular mortality between individual GLDs, using metformin monotherapy as reference.

**Results:**

The study comprised 9876 users of GLDs admitted with MI. The mean age was 72.3 years and 56.5% of patients were men. A total of 3649 received sulfonylureas and 711 received metformin at admission. The average length of follow-up was 2.2 (SD 2.6) years. A total of 6,171 patients experienced the composite study endpoint. The sulfonylureas glibenclamide, glimepiride, glipizide, and tolbutamide were associated with increased risk of cardiovascular mortality and/or nonfatal MI with hazard ratios [HRs] of 1.31 (95% confidence interval [CI] 1.17-1.46), 1.19 (1.06-1.32), 1.25 (1.11-1.42), and 1.18 (1.03-1.34), respectively, compared with metformin. Gliclazide was the only sulfonylurea not associated with increased risk compared with metformin (HR 1.03 [0.88-1.22]).

**Conclusions:**

In patients with diabetes mellitus admitted with MI not treated with emergent percutaneous coronary intervention, monotherapy treatment with the sulfonylureas glibenclamide, glimepiride, glipizide, and tolbutamide was associated with increased cardiovascular risk compared with metformin monotherapy.

## Introduction

Patients with diabetes mellitus (DM) have increased risk of cardiovascular disease (CVD) and worse outcomes after surviving an adverse cardiovascular event [1-3]. Prevention and management aimed at reducing CVD in DM include lifestyle interventions, cardiovascular pharmacotherapy (treatment of hypertension, dyslipidemia etc.), and glucose-lowering drugs (GLDs) [4]. Whether intensive control of blood glucose levels improves CVD has been intensively studied during the past few years and the optimum pharmacological treatment to reduce hyperglycaemia and CVD has not been established [5-10]. In particular, the report from the University Group Diabetes Program (UGDP) almost 40 years ago of increased cardiovascular mortality in DM patients receiving the first-generation sulfonylurea tolbutamide has not been conclusively refuted [11,12]. In contrast to the UGDP results, however, the UK Prospective Diabetes Study (UKPDS) found no increased mortality with sulfonylureas compared to conventional treatment primarily with diet alone, but suggested reduced risk of MI, stroke, and total mortality with metformin compared to treatment with diet, sulfonylurea or insulin, in obese patients with type 2 DM [13]. A more recent 10-year follow-up of UKPDS even suggested that sulfonylureas and insulin may be associated with reduced CVD comparable to the effects of metformin [14].

Sulphonylureas may inhibit myocardial preconditioning and theoretically, this detrimental effect may be of more importance in patients with MI undergoing emergent (primary) percutaneous coronary intervention (PCI) [15,16]. Furthermore, on-going treatment with metformin has been considered as a contraindication for intravenous contrast exposure (e.g., during emergent PCI) because of a perceived risk of lactic acidosis, although this contention is increasingly debated [17,18]. In view of these uncertainties regarding the safety of oral GLDs in patients with MI, we recently examined long term outcomes in diabetes patients with MI that underwent emergent PCI, and found that glibenclamide, but not other sulphonylurea agents, was associated with increased mortality and morbidity compared to metformin [19]. Patients undergoing emergent PCI only represent a fraction of the MI population, and the present study was therefore undertaken to investigate effects of oral GLDs on long term outcomes in patients with MI that did not undergo emergent PCI.

## Materials and methods

### National registers

In Denmark, all citizens have a unique personal civil registration number that enables individual linkage of information across nationwide registers. The National Patient Register keeps information of all admissions and invasive therapeutic procedures performed in Danish hospitals since 1978. Each admission is registered by a primary, and, if appropriate, one or more secondary diagnoses, coded according to the 10^th ^revision of the International Classification of Diseases (ICD-10). The diagnosis of acute MI has previously been validated in the National Patient Registry with a specificity of 93% [20]. The Danish Register of Medicinal Product Statistics (National Prescription Register) holds information on all prescriptions dispensed from Danish pharmacies since 1995. Drugs are registered according to the international Anatomical Therapeutical Chemical (ATC) classification. Because of the national health care reimbursement scheme of drug expenses, pharmacies in Denmark are required to register all dispensed prescriptions ensuring complete registration [21]. The civil registration system holds information on vital status for all citizens, and all deaths are registered within 14 days of occurrence. Primary and contributing causes of mortality are registered in the National Causes of Mortality Register according to the ICD-10.

### Study population and GLDs

Patients aged 30 years or older admitted to Danish hospitals during 1997-2006 with a diagnosis of MI (ICD-10 codes I21-I22) were identified from the National Patient Registry. In this study, we included all patients admitted with MI that did not undergo percutaneous coronary intervention (PCI) procedures during the first 48 hours after admission. PCI was detected by use of the Danish Health Care Classification System codes KFNG02 and KFNG05. Patients with DM were identified as individuals claiming at least one prescription for GLDs (oral agents or insulin) in the period ≤ 180 days prior to admission with MI. Treatment received in this period was defined as baseline treatment and used as a fixed variable in the analyses. By determination of the amount of dispensed tablets, their strengths, and dispensing time intervals for up to 7 consecutively claimed prescriptions after discharge, an individually adjusted coverage of drug treatment at the time of a study endpoint (see below) was calculated for all GLDs. This method has been described in details previously [22]. Patients with DM were subdivided into groups receiving specific GLD monotherapies, combinations of two or more oral agents and/or insulin, or no GLD, respectively. All claimed prescriptions for insulin (ATC A10A), metformin (ATC A10B), glimepiride (ATC A10BB12), glibenclamide, known as glyburide in the U.S. and Canada (ATC A10BB01), glipizide (ATC A10BB07), gliclazide (ATC A10BB09), and tolbutamide (ATC A10BB03) were included. DM patients on dietary treatment alone or patients that did not claim any prescriptions for GLDs ≤ 180 days prior to the admission with MI were not included. Patients requiring insulin monotherapy are more likely to have type 1 DM and a more advanced stage of diabetes compared with patients receiving oral GLDs. We therefore included patients treated with insulin both to test the a priori assumption that these subjects would have the greatest risk of CVD, but also to allow for identification of patients that changed to insulin during the follow up period and to allow for indiscriminate detection of all GLDs. Only 12 patients received alpha-glucosides in monotherapy and they were therefore excluded from the analysis. Thiazolidinediones are not recommended as monotherapy in Denmark and during the study period no patients were identified receiving these drugs in monotherapy.

### Concomitant pharmacotherapy, comorbidity and socioeconomic status

All variables listed below were selected a priori. We examined concomitant cardiovascular pharmacotherapy as determined by prescriptions claimed ≤ 180 days prior to the index MI to describe concomitant pharmacotherapy at baseline and furthermore prescriptions claimed ≤ 90 days after the index admission. The following drugs were identified: angiotensin converting enzyme (ACE) inhibitors and angiotensin-2 receptor blockers (ATC C09), β-blockers (ATC C07) spironolactone (ATC C03D), loop diuretics (ATC C03C), clopidogrel (ATC B01AC04), vitamin K-antagonists (ATC B01AA), and statins (ATC A10A). Concomitant pharmacotherapy was used as fixed variables in both types of analyses performed. Co-morbidity was defined according to the modified Ontario Myocardial Infarction Mortality Prediction Rules by diagnoses from the index admission and 1 year prior to admission (ICD-10 codes) [23]. The diagnoses used were congestive heart failure, cardiac dysrhythmias, pulmonary oedema, shock, cerebrovascular disease, DM with chronic complications, acute renal failure, chronic renal failure, and cancer. Socioeconomic status was defined by the individual average yearly gross income during a 5-year period before the index MI. Patients were divided into quartiles according to their income.

### Endpoints

The primary endpoint of the study was a composite of nonfatal MI (ICD-10 codes I21-I22) and cardiovascular mortality, defined as death with CVD (ICD-10 codes I00-I99) listed as a primary or contributing cause of death. Secondary endpoints were cardiovascular mortality and all-cause mortality, respectively. Nonfatal MI was defined as a subsequent admission with MI more than 30 days after the index admission.

### Statistical Analysis

Multivariable Cox proportional-hazard regression analyses were used to asses risk with individual GLDs. Exposure to GLDs was included as a fixed variable for baseline treatment and time-dependent variable for analyses performed at time of endpoint. Further, time-dependents analyses of adverse CVD events after MI patients were also performed such that patients were only considered at risk when taking the respective GLD. All Cox models were censored for deaths resulting from causes not related to the endpoints of interest. For the composite endpoint, we estimated the time to the first event (nonfatal MI or cardiovascular mortality). Cox analyses were adjusted for age, sex, socioeconomic status, calendar year, concomitant pharmacotherapy and co-morbidity. For all analyses, metformin was used as reference. The proportional-hazard assumption was tested specifying both the survival function versus time, as well as the log(-log(survival) versus log (time) showing parallel curves satisfying the assumption [24]. The continuous variables age and lifetime were also tested and found to be linear. The models were tested for lack of interactions and found to be valid. All statistical calculations were performed with the SAS statistical software package, version 9.1 (SAS Institute Inc, Cary, North Carolina).

## Ethics

The Danish Data Protection Agency approved the study (No. 2007-41-1667). Retrospective register studies where data are processed in a form where individuals can not be identified, do not require ethical approval in Denmark [25].

## Results

During the study period (1997-2006), 94,808 patients where admitted with MI of which 10,808 claimed glucose-lowering drugs < 180 days prior to the date of admission. After exclusion of patients receiving emergent PCI during the first 2 days of admission, the final study population consisted of 9,876 patients. The baseline characteristics of the cohort are presented in Table 1. The mean age at admission was 72.3 years and 56.5% of patients were men. The average length of follow-up was 2.2 (SD 2.6) years. By calculating the time from first claimed prescription of GLDs to inclusion in the study with MI, the mean duration of diabetes was 4.8 years (SD 3.0), although information on medical therapy was only available from 1995 and onwards. Factors known to affect mortality, such as age, sex and co-morbidity were similar in the GLD exposure groups except for patients receiving metformin, who were younger, had less congestive heart failure, pulmonary oedema, and chronic renal failure, and were more likely to receive statins compared with patients treated with sulfonylureas. Comparisons of patients treated with individual sulfonylureas showed that patients receiving gliclazide were younger and patients receiving tolbutamide were older and had more cerebrovascular disease.

**Table 1:** Baseline characteristics

	Total	Metformin	Insulin	Glibenclamide	Glimepiride	Glipizide	Gliclazide	Tolbutamide	Combo*
(%)	9876	711(7.2)	2889(29.3)	1136(11.5)	1180(12.0)	569(5.8)	221(2.2)	543(5.5)	2615(26.5)
Age (mean [SD)	72.3(11.2)	68.7(11.4)	70.5(12.1)	75.3(10.3)	74.7(10.5)	75.6(10.2)	73.9(10.4)	76.1(10.6)	71.4(10.5)
Men (%)	5582 (56.5)	439(7.9)	1476(26.4)	666(11.9)	712(12.8)	323(5.8)	134(2.4)	300(5.4)	1522(27.3)
Age; men (mean [SD)	70.3(11.2)	66.8(11.0)	68.2(12.0)	73.1(10.6)	72.7(10.6)	73.5(10.6)	72.4(11.0)	73.8(11.1)	69.6(10.3)
Women (%)	4294(43.5)	272(6.3)	1413(32.9)	470(11.0)	468(10.9)	246(5.7)	87(2.0)	243(5.7)	1093(25.5)
Age; women (mean [SD)	75.0(10.7)	71.9(11.2)	73.0(11.8)	78.4(9.1)	77.9(9.6)	78.4(8.9)	76.0(9.0)	78.9(9.1)	73.9(10.1)
**Study inclusion period**									
1997-1998	2034(20.6)	57(2.8)	566(27.8)	410(20.2)	56(2.8)	207(10.2)	43(2.1)	213(10.5)	482(23.7)
1999-2000	1898(19.2)	84(4.3)	557(29.4)	273(14.4)	200(10.5)	157(8.3)	44(2.3)	128(6.7)	451(23.8)
2001-2002	2310(23.4)	175(7.6)	684(29.6)	229(9.9)	328(14.2)	111(4.8)	44(1.9)	116(5.2)	620(26.8)
2003-2004	2031(20.6)	203(10.0)	581(28.6)	153(7.5)	349(17.2)	61(3.00)	61(3.00)	57(2.8)	564(27.8)
2005-2006	1603(16.2)	192(12.0)	501(31.3)	71(4.4)	247(15.4)	33(2.1)	29(1.8)	29(1.8)	498(31.1)
**Comorbidity**									
Congestive heart failure	1963(19.9)	104(14.6)	621(21.5)	205(18.1)	239(20.3)	101(17.8)	44(19.9)	113(20.8)	536(20.5)
Cardiac dysrhythmias	1264(12.8)	81(11.4)	381(13.2)	134(11.8)	175(14.8)	66(11.6)	36(16.3)	72(13.3)	317(12.1)
Pulmonary oedema	332(3.4)	13(1.8)	99(3.4)	51(4.5)	19(1.6)	21(3.7)	6(2.7)	30(5.5)	93(3.6)
Shock	286(2.9)	14(2.0)	111(3.8)	27(2.4)	32(2.7)	17(3.0)	5(2.3)	12(2.2)	68(2.6)
Cerebrovascular disease	927(9.4)	51(7.2)	302(10.5)	85(7.5)	116(9.8)	45(7.9)	16(7.2)	67(12.3)	244(9.3)
Diabetes mellitus with chronic complications	3830(38.8)	193(27.1)	1627(56.3)	26662(23.1)	364(30.9)	142(25.0)	62(28.1)	151(27.8)	1023(39.1)
Acute renal failure	213(2.2)	13(1.8)	108(3.7)	21(1.9)	13(1.1)	10(1.8)	5(2.3)	9(1.7)	34(1.3)
Chronic renal failure	267(2.7)	7(1.0)	150(5.2)	23(2.0)	33(2.8)	7(1.2)	3(1.4)	11(2.0)	33(1.3)
Cancer	465(4.7)	22(3.1)	162(5.6)	61(5.4)	64(5.4)	35(6.2)	9(4.1)	18(3.3)	92(3.5)
**Concomitant medication**									
ACE **^† ^**inhibitors or ARBs **^‡^**	4515(45.7)	344(48.4)	1543(53.4)	354(31.2)	518(43.9)	195(34.3)	90(40.7)	173(31.9)	1291(49.4)
β-Blockers	2319(23.5)	181(25.5)	642(22.2)	273(24.0)	315(26.7)	125(22.0)	51(23.1)	97(17.9)	635(24.3)
Oral anticoagulants	591(6.0)	40(5.6)	171(5.9)	43(3.8)	84(7.1)	23(4.0)	23(10.4)	30(5.5)	176(6.7)
Loop diuretic agents	4188(42.4)	208(29.3)	1486(51.4)	416(36.6)	466(39.5)	216(38.0)	87(39.4)	216(39.8)	1092(41.8)
Spironolactone	783(7.9)	49(6.9)	267(9.2)	48(4.2)	107(9.1)	36(6.3)	15 (6.8)	29(5.3)	230(8.8)
Statins	2031(20.6)	218(30.7)	609(21.1)	106(9.3)	260(22.0)	53(9.3)	34(15.4)	50 (9.2)	697(26.7)

*Combo = combinations of two or more GLDs. **^†^**ACE = angiotensin converting enzyme **^‡^**ARB = angiotensin II receptor blockers. Only patients receiving monotherapy at baseline at the time of a study end point were examined. Patients receiving insulin and combination treatment were included in the table allowing for changes in treatment during the study period. 12 patients received acarbose treatment and were excluded from further analysis.

### Mortality and nonfatal MI

**Figure 1:**
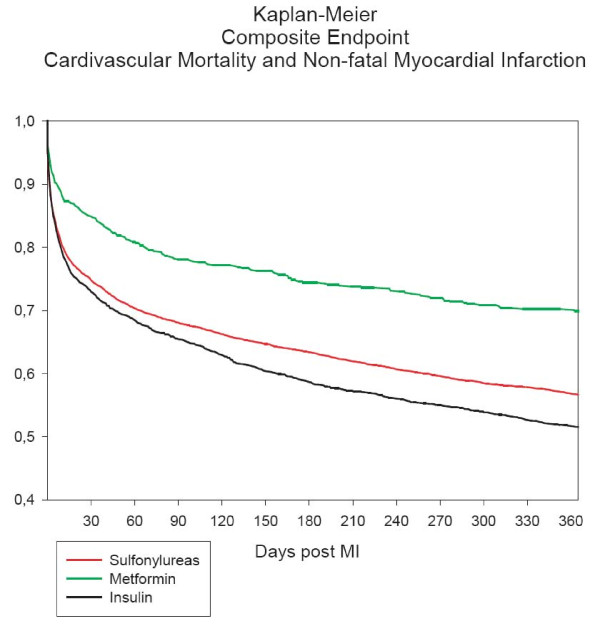
**Kaplan-Meier curves for the composite endpoint of cardiovascular mortality and non-fatal myocardial infarction (MI) one year after index MI**. Treatment groups were baseline glucose-lowering drug monotherapy ≤ 180 days prior to MI.

**Figure 2:**
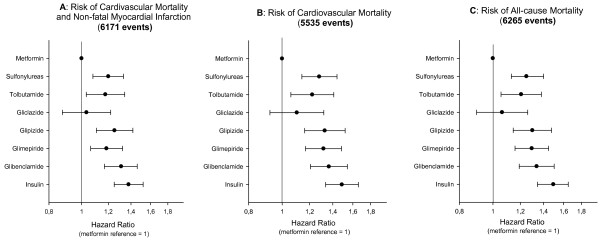
**A: Risk of cardiovascular mortality and non-fatal myocardial infarction (MI), B: risk of cardiovascular mortality, and C: risk of all-cause mortality associated with glucose-lowering drug monotherapy**. Time-dependent Cox analyses were performed with adjustments for age, sex, socioeconomic status, calendar year, concomitant medication and co-morbidity. Analyses for both individual sulfonylureas and sulfonylureas grouped as one pharmacological class were performed.

Kaplan-Meier analyses were used to calculate survival probability estimates of 0.440, 0.366, and 0.375 for cardiovascular mortality, all-cause mortality, and rates of nonfatal MI and cardiovascular mortality, respectively, for the entire study period. Figure 1 shows Kaplan-Meier curves for GLD treatment received as monotherapy ≤ 180 days prior to MI for the composite endpoint of non-fatal MI and cardiovascular mortality one year post MI, with all sulfonylureas grouped as one pharmacologic class. Time-dependent Cox proportional-hazards regression analyses only having patients at risk while using medication showed increased risk of cardiovascular mortality, nonfatal MI and cardiovascular mortality, and all-cause mortality with glibenclamide, glimepiride, glipizide, and tolbutamide, compared with metformin (Table 2). As indicated in Figure 2A,B and 2C, gliclazide was not associated with increased risk compared with metformin.

**Table 2:** Hazard ratios for studied end points according to treatment at time of a study endpoint

Glucose-lowering drug	Cardiovascular mortality		Composite of cardiovascular mortality and non-fatal MI		All-cause mortality	
	HR	95% CI	HR	95% CI	HR	95% CI
Metformin	1		1		1	
Insulin	1.49	1.34-1.66	1.38	1.25-1.52	1.50	1.35-1.65
Glibenclamide	1.37	1.21-1.54	1.31	1.17-1.46	1.34	1.19-1.50
Glimepiride	1.32	1.17-1.48	1.19	1.06-1.32	1.30	1.16-1.45
Glipizide	1.33	1.16-1.52	1.25	1.11-1.42	1.30	1.14-1.48
Gliclazide	1.10	0.92-1.32	1.03	0.88-1.22	1.06	0.90-1.26
Tolbutamide	1.22	1.06-1.41	1.18	1.03-1.34	1.21	1.06-1.38
All sulfonylureas	1.28	1.14-1.44	1.20	1.08-1.33	1.25	1.13-1.40

Hazard ratios (HRs) and 95% confidence intervals (CIs) for adverse cardiovascular end points according to glucose-lowering drug (monotherapy) at the time of a study endpoint. Time-dependent Cox analyses were performed with adjustments for age, sex, socioeconomic status, calendar year, concomitant pharmacotherapy, and comorbidity. MI = Myocardial infarction.

Comparisons of hazard ratios (HRs) with individual sulfonylureas at baseline and at the time of a study endpoint, respectively, showed similar results for all GLDs apart from glimepiride and tolbutamide. Glimepiride was associated with greater risk when analyzed at the time of endpoints and tolbutamide was exclusively associated with increased all-cause mortality when analyzed at the time of a study endpoint. Insulin treatment was associated with the greatest risk for all endpoints (Table 2 and 3). Treatment guidelines changed during the study period and additional analyses were performed only including patients included after January 31, 2001, to identify whether time of admission would compromise the results found for the entire period. No statistically significant interactions with individual GLD treatments and time of admission were found.

**Table 3:** Hazard ratios for studied end points according to treatment at baseline

Glucose-lowering drug	Cardiovascular mortality		Composite of cardiovascular mortality and non-fatal MI		All-cause mortality	
	HR	95% CI	HR	95% CI	HR	95% CI
Metformin	1		1		1	
Insulin	1.47	1.29-1.68	1.46	1.29-1.66	1.47	1.30-1.66
Glibenclamide	1.31	1.13-1.51	1.34	1.17-1-54	1.26	1.10-1.44
Glimepiride	1.15	1.00-1.33	1.15	1.01-1.32	1.14	1.00-1.31
Glipizide	1.19	1.01-1.40	1.24	1.06-1.44	1.18	1.01-1.37
Gliclazide	1.04	0.83-1.30	1.09	0.89-1.35	1.00	0.81-1.23
Tolbutamide	1.19	1.01-1.40	1.22	1.05-1.43	1.16	0.99-1.35
All sulfonylureas	1.20	1.05-1.37	1.22	1.08-1.38	1.17	1.04-1.32

Hazard ratios (HRs) and 95% confidence intervals (CIs) for adverse cardiovascular end points according to glucose-lowering drug (monotherapy) at baseline. Time-dependent Cox analyses were performed with adjustments for age, sex, socioeconomic status, calendar year, concomitant pharmacotherapy, and comorbidity. MI = Myocardial infarction.

The use of metformin and sulfonylureas before and after MI was very consistent. For patients receiving metformin prior to MI, 82.4% of the survivors claimed a new prescription for metformin. For patients receiving sulfonylureas, 94.2% of survivors claimed a new prescription for a sulfonylurea.

## Discussion

The current study is, to the best of our knowledge, the first to compare on a nation-wide scale the outcomes after MI in patients with DM treated with different GLDs that did not undergo emergent PCI. We found that after MI, patients treated with selected sulfonylureas including glibenclamide, glimepiride, glipizide, and tolbutamide had increased risk of cardiovascular mortality and nonfatal MI, cardiovascular mortality, and all-cause mortality, respectively, compared to patients receiving metformin. Gliclazide was the only sulfonylurea consistently not associated with increased risk of adverse cardiovascular events. The results contribute to data indicating that sulphonylureas may be associated with adverse cardiovascular events in patients with MI and they suggest that metformin should generally not be discontinued in these patients.

### Sulphonylureas: Pharmacological effects and tissue specificities

The primary pharmacological action of sulfonylureas is closure of K_ATP _channels in the pancreatic β-cell membrane, which depolarizes the cell and triggers insulin release. However, K_ATP _channels in coronary vascular smooth muscle cells and cardiomyocytes play important roles in control of the coronary blood flow and the signalling cascades underlying myocardial ischemic preconditioning [15,26,27]. In this regard, the affinity of individual sulfonylureas for pancreatic and cardiac K_ATP _channels differs substantially. For example, glibenclamide in clinically relevant concentrations can reduce myocardial protection afforded by preconditioning, whereas newer sulfonylureas such as glimepiride and gliclazide do not appear to display these cardiac effects [28-34]. Indeed, glibenclamide (but not other sulfonylureas) was therefore recently specifically omitted in a consensus treatment algorithm for initiation and adjustment of combined pharmacologic treatment for hyperglycaemia in patients with DM [35], and consistent with these results, glibenclamide was the sulfonylurea associated with the greatest risk in our study. In agreement with the current results, we recently reported that in DM patients with MI that underwent emergent PCI, i.e., the complimentary diabetic MI population for the present study, glibenclamide was associated with increased risk of cardiovascular mortality, cardiovascular mortality and nonfatal MI, and all-cause mortality, respectively, compared to patients receiving metformin [17]. In that study, the HR for the composite of cardiovascular death and nonfatal MI with glibenclamide compared to metformin was approximately twice the HR found in the current study, while other sulphonylurea drugs were not associated with increased risk, although significant heterogeneity between individual agents was observed [17]. At present, therefore, it is unclear whether adverse cardiovascular outcomes associated with glibenclamide in DM patients with MI, represent a pharmacological class effect of sulphonylureas. Gliclazide is highly selective for pancreatic K_ATP _channels and was not associated with increased risk of adverse cardiovascular events in the present study. Interestingly, disparate cardiac effects of glibenclamide and gliclazide on myocardial preconditioning have been demonstrated in patients with stable angina pectoris, where increased exercise tolerance conferred by exercise-induced ischemic preconditioning was abolished by glibenclamide, but was somewhat preserved with gliclazide [32]. Moreover, experimental studies have indicated that gliclazide may prevent or retard the development of atherosclerosis by exerting antioxidant effects and by reducing monocyte adhesion to vascular cells [36]. We found that tolbutamide was associated with increased risk compared to metformin but the risk with tolbutamide was not significantly different from that of treatment with gliclazide (p > 0.05 for all endpoints). Tolbutamide and gliclazide appear to be highly pancreatic β-cell-selective compared with glimepiride and glibenclamide [27]. Our finding of similar risk associated with tolbutamide and gliclazide therefore supports the hypothesis that the differences in outcomes after MI with different sulfonylureas may be explained, in part, by differences in K_ATP _channel tissue specificities.

### Metformin vs. sulphonylureas

The therapeutic effects of metformin and sulfonylureas are mediated through entirely different mechanisms. The glucose-lowering effects of metformin are mainly a consequence of reduced hepatic gluconeogenesis and increased insulin-stimulated glucose uptake in skeletal muscle and fat tissue [37]. Metformin appears to improve vascular function, exert favourable effects on lipid metabolism, and reduce hypercoagulability and platelet reactivity in patients with DM [37]. These effects may have contributed to the improved cardiovascular mortality observed here in patients receiving metformin compared with sulfonylureas. After MI, patients may be advised to change GLDs, e.g., discontinue metformin because of concern for risk of lactic acidosis, congestive heart failure, and renal impairment, or discontinue sulfonylureas due to a perceived risk of impairing myocardial preconditioning. Our current results in patients with MI that did not undergo PCI within the first48 hours after admission, however, showed that the use of GLDs was relatively consistent before and after the event, indicating that physicians and patients generally adhere to their established GLDs and are not markedly influenced by the aforementioned concerns. Importantly, no adverse cardiovascular endpoints were associated with continuation of metformin in these patients.

A recently published study comparing outcomes with sulfonylureas and metformin in patients with DM included in a large UK general practice research database found that sulfonylureas were associated with increased risk of first-time MI and all-cause mortality [38]. This increased risk, however, was not statistically significant after adjustments for multiple confounders possibly because of a relatively small sample size. In our study, we exclusively analyzed data from individuals with complete information on all examined variables, and we consistently found an increased risk with sulfonylureas regardless of adjustments for covariates. In aggregate, the evidence therefore suggests an unfavourable cardiovascular risk profile of sulfonylureas compared with metformin.

### Strengths and limitations

The main strength of this study was the nationwide consecutive patient registries, the contemporary data collection, the large sample comprising nearly 10,000 patients, the assessment of important clinical outcomes, and the use of pharmacy dispensations and not prescriptions alone, i.e., with the latter better reflecting true as opposed to intended drug use. We hereby avoided selection bias otherwise arising from inclusion of only subgroups of patients or patients from selected hospitals, medical centres, or health care systems. Furthermore, the cohort comprised patients both in and out of the labour market. In Denmark, a government-financed health care system ensures free and equal access to health care for all inhabitants. The main study limitation is inherent to the observational nature of the study design. Although appropriate adjustments were made for comorbidity, socioeconomic factors and co-medication, the effect of unmeasured confounders cannot be excluded. The registers did not include information on the exact level of drug adherence and other clinical characteristics, e.g., presence of preinfarction angina, blood glucose levels, exact diabetes duration, cholesterol levels, smoking habits and body mass index, that could have given a more detailed picture of patient risk profiles. Furthermore, the codes in the National Patient Registry cannot discriminate between MI with or without ST segment elevation. Although primary PCI was quickly established on a national scale after 2002-3 for patients with ST segment elevation MI following the results of the Danish Multicenter Randomized Study on Fibrinolytic Therapy versus Coronary Angioplasty in Myocardial Infarction (DANAMI-2) trial [39], MI patients in the present study were a mixture of subjects with or without ST segment elevation on the presenting ECG, and the ratio of these two MI types changed during the study period, i.e., with a larger fraction of patients with ST segment elevation MI that received fibrinolytic treatment on admission in the early study period, and a predominant population of non-ST segment MI patients in the later study period. Specific effects of GLDs in MI patients with or without ST segment elevation therefore require further investigation, but it is notable that the results of our previous study of outcomes with different GLDs in MI patients that underwent emergent PCI were comparable to the current data [17]. We also cannot exclude the possibility that the slightly higher proportion of patients discontinuing metformin as opposed to sulfonylurea after MI may preferentially have included those with the most severely reduced left ventricular function and adverse prognosis, but it seems highly unlikely that such confounding would significantly influence our results. It should also be noted that the study cohort was homogeneous in terms of ethnicity and our findings may not be applicable to other populations. Moreover, metformin is the advocated drug of choice in overweight patients with type 2 DM and it is possible that it was given preferentially to patients with milder disease. Finally, DM in itself may compromise the capacity for preconditioning and the adverse cardiovascular effects of the analyzed sulfonylureas may have been dependent on other mechanisms than inhibition of preconditioning [40,41].

## Conclusions

In conclusion, the current study of cardiovascular outcomes with different GLDs in DM patients with MI that did not undergo emergent PCI showed that glibenclamide, glimepiride, glipizide, and tolbutamide were associated with increased risks of cardiovascular mortality and nonfatal acute MI, cardiovascular mortality, and all-cause mortality, respectively, compared to treatment with metformin. Gliclazide was not associated with increased risk of adverse cardiovascular events compared to metformin. Different affinities of individual sulphonylureas for pancreatic and cardiac K_ATP _channels may have contributed to these findings. Further studies of the cardiovascular safety of sulphonylureas are warranted and the results indicate that metformin should not be discontinued following MI in patients with DM.

## Competing interests

AV Works at Steno Diabetes Center, which is owned by Novo Nordisk. Apart from this there are no relationships to disclose.

## Authors' contributions

CJ, GG, SA CTP and PH conceived and designed the study. CJ and GG acquired and analyzed data. CJ drafted the first version of the manuscript. All authors interpreted data and critically revised the manuscript for important intellectual content and accepted the final version of the manuscript for submission.
